# Use of Systematic Review and Meta-Analysis in Environmental Health Epidemiology: a Systematic Review and Comparison with Guidelines

**DOI:** 10.1007/s40572-015-0062-z

**Published:** 2015-07-03

**Authors:** Mary C. Sheehan, Juleen Lam

**Affiliations:** Department of Health Policy and Management, Johns Hopkins Bloomberg School of Public Health, 615 North Wolfe Street, Baltimore, MD 21205 USA

**Keywords:** Systematic review, Meta-analysis, Environmental health, Reporting guidelines, Air pollution, Environmental epidemiology

## Abstract

Systematic review (SR) and meta-analysis (MA) have potential to contribute substantially to environmental health (EH) risk assessment and policy-making, provided study questions are clear and methods sound. We undertook a systematic review of the published epidemiological literature for studies using both SR and MA examining associations between chronic low-dose chemical exposures and adverse health outcomes in general populations and compared actual methods and reporting with a checklist based on available published guidelines. We identified 48 EH SRMAs meeting these criteria. Associations were mainly positive and statistically significant, often involving large populations. A majority of studies followed most general SRMA guidance, although we identified weaknesses in problem formulation, study search, selection and data extraction, and integrating policy implications. Fewer studies followed EH-specific SRMA recommendations, particularly regarding exposure heterogeneity and other risks of bias. Development and adoption of EH-specific SRMA guidelines would contribute to strengthening these tools for public health decision-making.

**Electronic supplementary material:**

The online version of this article (doi:10.1007/s40572-015-0062-z) contains supplementary material, which is available to authorized users.

## Introduction

The World Health Organization (WHO) estimates that environmental factors are responsible for at least one quarter of the global burden of disease [1]. A growing body of evidence suggests a considerable share stems from exposure to environmental chemicals [2]; in many cases, such exposures are widespread across large populations or involve vulnerable groups [2, 3]. Preventing environmentally related disease requires the translation of up-to-date scientific research into accessible evidence that informs environmental health (EH) risk assessment, policy formulation, health care practice, and individual health behaviors [4, 5]. However, the scientific literature is diverse, of varying quality, and often not easily accessible to decision-makers [6].

The tools of evidence-based science — systematic review (a literature review that poses a well-defined and specific research question, uses systematic and explicit methods to identify, select and appraise research, and analyze data from selected studies) and meta-analysis (a quantitative statistical analysis that integrates results of chosen studies) [7] — are well-developed for randomized controlled trials and making recommendations in clinical medicine. These tools also have the potential to play a role in EH risk assessment and policy-making. When used together, systematic review and meta-analysis (SRMA) techniques provide rigorous rules designed to gather all relevant research on a well-defined study question, statistically pool outcomes across studies with comparable methods, and provide a transparent, thorough, and replicable examination of available evidence. This can help to overcome the drawbacks of small sample size, demonstrate where effects are consistent across studies and generalizable across populations and where they are not, minimize bias and reduce chance effects, and identify research gaps [8]. EH science evidence with these features can help facilitate efficient policy-making.

Human epidemiological studies are the preferred body of evidence for EH, as they represent real-world background and co-exposures and do not require species extrapolation. However, in practice, the observational designs of human epidemiological studies in EH pose challenges to conducting MA that have contributed to slower adoption of SRMA. Among these are inability to fully control for confounders at all times, inconsistencies across studies in exposure measurement methods and metrics, and differences in outcomes, populations, and study designs [9]. Development of special meta-analytical methods to control for confounding [10] and risk-of-bias assessment checklists [11] have contributed to improving SRMA methods for observational studies. Codification of reporting guidance, such as the Meta-Analysis of Observational Studies in Epidemiology (MOOSE) consensus statement [12], has also advanced the field; use of such guidelines is associated with higher quality reporting [13]. More recently, the US National Institutes of Environmental Health Sciences (NIEHS) National Toxicology Program (NTP) has adopted SRMA in its chemical assessments [14•]; the US EPA is examining using these tools for risk assessment under its Integrated Risk Information System (IRIS) program [15•]; and the Navigation Guide group (an interdisciplinary collaboration between academicians, practitioners, and clinicians) has developed a framework to assess the strength of EH science evidence [16, 17•]. These newer initiatives are contributing to the refinement and codification of methodological approaches for SRMA tailored to the specificities of EH.

The potential of SRMA to contribute to EH policy, the development of more robust, EH-specific methods, and growing interest in their use suggest the need to take stock of the nature and quality of the current body of published EH SRMAs. We undertook a systematic review of the EH literature to identify SRMA studies examining associations between chronic low-dose chemical exposures and adverse health outcomes in general human populations in order to compare methods used with available published consensus reporting guidelines. The broad goal of this review is to contribute to enhancing the utility and expanding use of these powerful evidence-synthesis tools for EH policy.

## Methods

We conducted a search of the published scientific literature using Medline, Embase, Web of Science, Scopus, and Google Scholar (with the search terms “environment” OR “pollutant” OR “contaminant” AND “disease” OR “chronic AND disease” OR “health AND effects” AND “systematic” AND “review” AND “meta-analysis” OR “meta AND analysis” OR “quantitative AND analysis,” screened for human studies only). We performed topic searches in these electronic databases examining keywords, titles, and abstracts for designated terms and also hand-searched reference lists of selected studies. We did not restrict by language or starting date. Our search end date was June 30, 2013. We screened all titles and abstracts identified and reviewed full texts of articles that met prespecified inclusion criteria. We included only studies employing both SR and MA. We did not include SRs done without MA (e.g., SRs done as scoping reviews, SRs without quantitative outcomes, or SRs where reported outcomes were inadequate for a statistical analysis) or MAs for which SR had not been conducted (e.g., MA used to combine results across multicenter epidemiological studies or dose-response assessment using MA based on a few specifically chosen studies rather than a SR). We included SRMAs examining general, non-occupationally exposed populations exposed to chemicals at chronic, low dose (excluding biological, mental, or physical exposures, accidental high-dose acute exposures, intentional exposures such as alcohol or tobacco, and secondhand smoke exposure) that examined associations with one or more adverse health effects.

Both authors independently extracted data from included studies (differences were resolved by consensus), using purpose-designed data extraction forms previously tested on a pilot group of reviews. Extracted data included study goal, population characteristics, environmental exposures and their measurement protocols, health outcomes and their ascertainment procedures, summary effect measures (with confidence bounds), and responses to a checklist of 61 parameters of study methodological and reporting quality. We developed the checklist based on guidelines for SRMAs available to researchers during our review period, including the Preferred Reporting Items for Systematic Reviews and Meta-Analyses (PRISMA) statement [7], the MOOSE statement [11], and guidelines for use of SRMA in environmental epidemiology resulting from a 1994 workshop (referred to as “Blair et al.”) [18]. Checklist items for the general features of SRMA were based primarily on the PRISMA and MOOSE statements, while those related to quality and risk-of-bias evaluation, heterogeneity testing, exposure measurement, and outcome ascertainment were also based on Blair et al. The checklist questions were designed to have up to five possible responses: yes (Y), consistent with guideline; partial (P), in some part consistent with guideline; no (N), inconsistent with guideline; cannot determine based on data (ND); and not applicable to study (NA). For each parameter, definitions corresponding to guideline recommendations were developed beforehand to facilitate consistency in reviewer data extraction (available in Supplement 1). Responses were coded and combined in Microsoft Excel and transferred to STATA version 10.0 (StataCorp, College Station, TX).

## Results

**Fig. 1:**
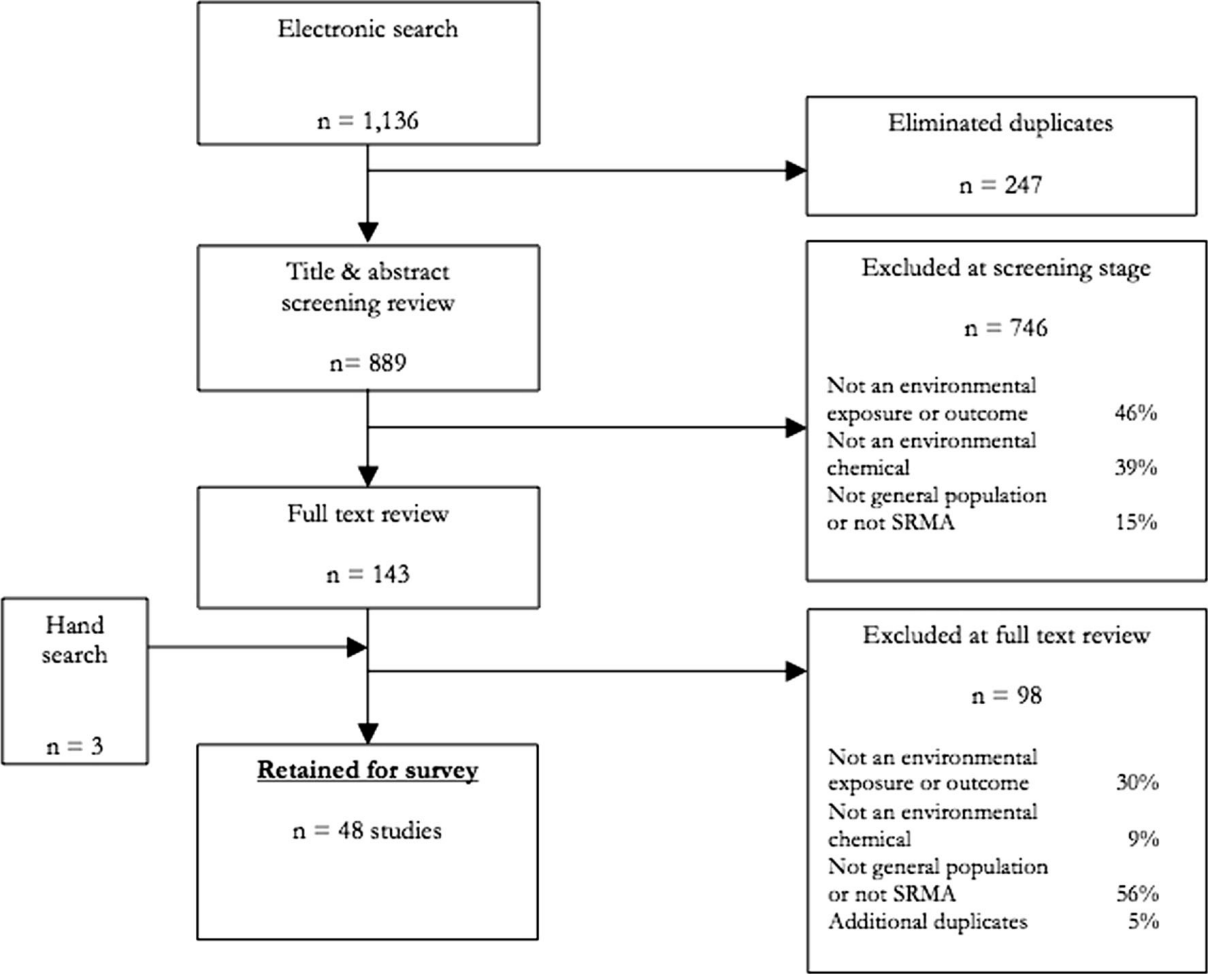
Systematic review of environmental health (EH) epidemiology systematic review and meta-analyses (SRMA): search and selection flow diagram

**Fig. 2:**
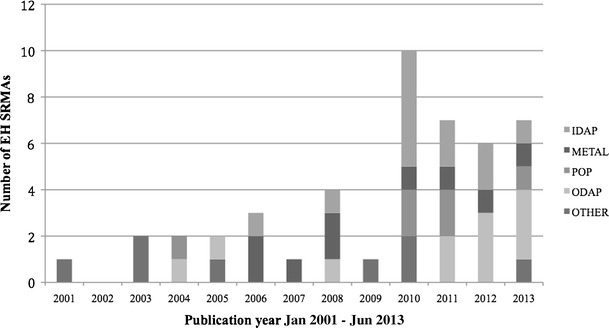
Systematic review of environmental health (EH) epidemiology systematic review and meta-analyses (SRMA): EH SRMAs by publication year and chemical category

Our search identified 1136 articles, of which the full texts for 146 were reviewed and 48 were selected (Fig. 1 and Table 1, details in Supplement 1). All except two SRMAs were published after 2000, the year the MOOSE guidelines were issued; two thirds were published after 2009, the year the PRISMA guidelines were published (Fig. 2). Selected reviews covered a total of 16 chemicals or classes of chemicals, in five categories: (i) 11 reviews of indoor air pollution (IAP), consisting of various types of solid fuel smoke [19–29]; (ii) 12 reviews of various outdoor air pollution (OAP) constituents [30–41]; (iii) 10 reviews of metals [42–51]; (iv) seven reviews of persistent organic pollutants (POPs) [52–58]; and (v) eight reviews of other chemicals [59–66].

**Table 1:** Systematic review of environmental health (EH) epidemiology systematic review and meta-analyses (SRMA): characteristics of selected studies

Study reference	Chemical	Category	Health outcome	Population	Pooled estimate^a^
Indoor air pollution (IAP)					
Zhao et al. 2006	Coal smoke	CANC	Lung	Adults	OR 2.66 (1.39, 5.07)
Hosgood et al. 2011	Coal smoke	CANC	Lung	Adults	OR 2.15 (1.61, 2.89)
Kurmi et al. 2012	Coal smoke	CANC	Lung	Adults	OR 1.82 (1.60, 2.06)
Dherani et al. 2008	Solid fuel smoke	RESP	Pneumonia	Children < 5	OR 1.78 (1.45, 2.18)
Kurmi et al. 2010	Solid fuel smoke	RESP	COPD	Adults	OR 2.80 (1.85, 4.00)
McGwin et al. 2010	Formaldehyde	RESP	Asthma	Children	OR 1.17 (1.01, 1.36)
Hu et al. 2010	Biomass smoke	RESP	COPD	Adults	OR 2.44 (1.79, 3.33)
Po et al. 2011	Biomass smoke	RESP	ARI	Children < 5	OR 3.53 (1.94, 6.43)
Misra et al. 2012	Solid fuel smoke	RESP	ARI	Children < 5	OR 2.51 (1.53, 4.10)
Sumpter et al. 2013	Solid fuel smoke	RESP	Tuberculosis	Adults	OR 1.30 (1.04, 1.62)
Pope et al. 2010	Solid fuel smoke	REPDEV	Low birth weight	Infants	OR 1.38 (1.25, 1.52)
Outdoor air pollution (OAP)					
Chen et al. 2008	PM_2.5_	CANC	Lung	Adults	RR 1.21 (1.10, 1.32)
Mustafic et al. 2011	PM_2.5_	CVD	Myocardial infarction	Adults	RR 1.03 (1.02, 1.04)
Pieters et al. 2012	PM_2.5_	CVD	Heart rate variability	Adults	% −2.44 (−3.76, −1.12)
Li et al. 2012	PM_2.5_	CVD	Stroke	Adults	RR 1.01 (1.00, 1.01)
Shah et al. 2013	PM_2.5_	CVD	Heart failure	Adults	RR 1.016 (1.008, 1.023)
Hoek et al. 2013	PM_2.5_	CVD	CVD mortality	Adults	RR 1.15 (1.04, 1.27)
Shang et al. 2013	PM_2.5_	RESP	Respiratory mortality	Adults	RR 0.51 (0.30, 0.73)
Ward et al. 2004	PM_2.5_	RESP	Peak expiratory flow	Children	% −0.144 (−0.243, −0.044)
Weinmayr et al. 2010	NO_2_	RESP	Asthma symptoms	Children	OR 1.031 (1.001, 1.062)
Vrijheid et al. 2011	NO_2_	REPDEV	Congenital anomalies	Infants	OR 1.20 (1.02, 1.42)
Stieb et al. 2012	PM_10_	REPDEV	Low birth weight	Infants	OR 1.10 (1.05, 1.15)
Ito et al. 2005	Ozone	OTHER	All-cause mortality	Adults	% 1.6 (1.1, 2.0)
Metals					
Chu et al. 2006	Arsenic	CANC	Bladder	Adults	Slope factor 3 × 10^−5^
Mink et al. 2008	Arsenic	CANC	Bladder	Adults	RR 1.11 (0.95, 1.30)
Navas-Acien et al. 2008	Lead	CVD	Hypertension	Adults	OR 1.04 (1.01, 1.07)
Gallagher et al. 2010	Cadmium	CVD	Diastolic BP	Women	Beta 1.84 (0.95, 2.74)
Abhyankar et al. 2011	Arsenic	CVD	Hypertension	Adults	OR 1.27 (1.09, 1.47)
Moon et al. 2012	Arsenic	CVD	Multiple CVD outcomes	Adults	RR 1.32 (1.05, 1.67)
Pocock et al. 1994	Lead	REPDEV	IQ point loss	Children < 5	−2.53 (−3.33, −1.73)
Rodriguez-Barranco et al. 2013	Arsenic	REPDEV	IQ point loss	Children < 5	−0.39 (−0.84, 0.06)
Navas-Acien et al. 2006	Arsenic	OTHER	Diabetes type 2	Adults	OR 2.52 (1.69–3.75)
Aminzadeh et al. 2007	Mercury	OTHER	Multiple sclerosis	Adults	OR 1.24 (0.96, 1.61)
Persistent organic pollutants (POPs)					
Lopez-Cervantes et al. 2004	DDE	CANC	Breast	Women	OR 0.97 (0.87, 1.09)
Turner et al. 2010	Pesticides	CANC	Child leukemia	Children < 5	OR 1.54 (1.13, 2.11)
Van Maele-Fabry et al. 2011	Pesticides	CANC	Child leukemia	Children < 5	OR 1.74 (1.37, 2.21)
Bailey et al. 2011	Pesticides	CANC	Child leukemia	Children < 5	OR 1.37 (1.00, 1.88)
Priyadarshi et al. 2001	Pesticides	OTHER	Parkinson’s disease	Adults	OR 1.85 (1.31, 2.60)
Allen et al. 2013	Pesticides	OTHER	Parkinson’s disease	Adults	OR 1.36 (1.05, 1.75)
Wu et al. 2013	PCBs	OTHER	Diabetes type 2	Adults	1.70 (1.28, 2.27)
Other					
Morris et al. 1992	DBP	CANC	Bladder	Adults	OR 1.21 (1.09, 1.34)
Villanueva et al. 2003	DBP	CANC	Bladder	Adults	OR 1.4 (1.2, 1.7)
Takkouche et al. 2005	Hair dye	CANC	Hemopoietic	Adults	RR 1.15 (1.05, 1.27)
Rahman et al. 2010	DBP	CANC	Rectal	Adults	RR 1.30 (1.06, 1.59)
Hwang et al. 2003	DBP	REPDEV	Any birth defects	Infants	OR 1.25 (1.11, 1.40)
Nieuwenhuijsen et al. 2009	DBP	REPDEV	Any birth defects	Infant	OR 1.17 (1.02, 1.34)
Grellier et al. 2010	THM	REPDEV	SGA	Infants	OR 1.01 (1.00, 1.02)
Choi et al. 2013	Fluoride	REPDEV	IQ point loss	Infants	Diff. −0.45 (−0.56, −0.35)

^a^Pooled effect sizes shown are the principal chemical exposure and health effect associations reported in reviews (where more than one association was examined, the association shown is that with the largest number of underlying studies or with the strongest identified association among those with similar numbers of studies). Fuller results are reported in Supplemental Material File 1. Estimates compare exposed with unexposed or higher with lower exposure groups, or provide risk per unit of pollutant

*ARI* acute respiratory infection, *CANC* cancer, *COPD* chronic obstructive pulmonary disease, *CVD* cardiovascular disease, *DDE* dichlorodiphenyldichloroethylene, *DBP* disinfection by-products, *IQ* intelligence quotient, *NO*_*2*_ nitrogen dioxide, *PCBs* polychorinated biphenyls, *PM*_*2.5*_ particulate matter < 2.5 μm, *REPDEV* reproductive/developmental, *RESP* respiratory, *SGA* small for gestational age, *THM* trihalomethane

Associations with 28 health outcomes were explored, in five categories: (i) cancers, including bladder, breast, colorectal, lung, prostate, and childhood leukemia (27 % of reviews); (ii) non-cancer respiratory diseases, including childhood asthma, childhood pneumonia, chronic obstructive pulmonary disease (COPD), tuberculosis, and acute respiratory infection (21 %); (iii) reproductive and development disorders, including birth defects, poor birth outcomes, and developmental neurotoxicity (19 %); (iv) cardiovascular disease (CVD), including hypertension, myocardial infarction, stroke, and heart rate variability (19 %); and (v) other health outcomes, including Parkinson’s disease, multiple sclerosis, and general mortality (14 %).

Adults were assessed in 63 % of SRMAs, while infants or children alone or in combination with women were the focus of 37 % of reviews. Most reviews did not limit geographic coverage; however, the majority of underlying study populations were from North America, Europe, and/or Asia. Thirty-eight percent of reviews examined primarily (but not exclusively) case-control studies, 27 % primarily cohort studies, 16 % primarily cross-sectional studies, 6 % time series and case-crossover studies, and 13 % examined a mix in which no study design was preponderant. Sixty percent of selected SRMAs reported pooled odds ratios (ORs); one quarter reported pooled relative risks (RRs), and the remainder reported other measures. Information on funding sources was provided in 67 % of SRMAs. Where this information was provided, reviews were sponsored either by academic organizations (63 %) or government bodies (37 %). Conflict of interest statements were provided by about half of studies; most of these declared no conflict.

### SRMA Findings by Chemical Category

#### Indoor Air Pollution

The IAP SRMAs examined exposures due to smoke from solid fuels (coal, wood, animal dung, crop wastes) used in cooking and heating, mainly in Africa, Latin America, and Asia, with one study examining formaldehyde exposure. Nearly three quarters examined respiratory outcomes, and one quarter investigated cancers (mainly lung cancer) or other outcomes (Table 1). Among statistically significant findings of the largest magnitude, three SRMAs examining coal smoke exposure and lung cancer in adults reported ORs of 2.15 (95 % CI 1.61, 2.89, *n* = 25) [20], 1.82 (95 % CI 1.60, 2.06, *n* = 23) [23], and 2.66 (95 % CI 1.39, 5.07, *n* = 8) [25]. Three reviews of solid fuel smoke exposure and COPD in adults found pooled ORs of 2.44 (95 % CI 1.79, 3.33, *n* = 15) [21], 2.80 (95 % CI 1.85, 4.00, *n* = 12) [22], and 2.40 (95 % CI 1.47, 3.93, *n* = 6) [25]. Two reviews of children <5 years old examining solid fuel smoke exposure and respiratory infection found combined ORs of 2.51 (95 % CI 1.53, 4.10, *n* = 9) [24] and 3.53 (95 % CI 1.94, 6.43, *n* = 10) [25], while one review of solid fuel smoke and childhood pneumonia found an OR of 1.79 (95 % CI 1.26, 2.21, *n* = 24) [19].

#### Outdoor Air Pollution

The OAP SRMAs examined exposures including carbon monoxide (CO), ozone (O_3_), sulfur dioxide (SO_2_), nitrogen dioxide (NO_2_), particulate matter <2.5 μm (PM_2.5_) and <10 μm (PM_10_) in various combinations in large urban areas. Over 40 % evaluated CVD outcomes, and the remainder respiratory, and reproductive and development outcomes (Table 1). Among statistically significant associations of largest magnitude, an incremental 10 μg/m^3^ of PM_2.5_ was associated with various cardiovascular outcomes, including cardiovascular mortality (RR 1.15; 95 % CI 1.04, 1.27; *n* = 10) [31]; heart failure (RR 1.02; 95 % CI 1.01, 1.02, *n* = 10) [36]; myocardial infarction (RR 1.03; 95 % CI 1.02, 1.04; *n* = 13) [34]; and lower heart rate variability (decrease of 2.44 %, 95 % CI 3.76 %, 1.12 %, *n* = 13) [35].

#### Metals

The metal SRMAs covered exposure to arsenic, lead, manganese, and mercury through various exposure pathways, mainly in the USA and Asia. Forty percent of reviews examined cardiovascular outcomes and the remainder a range of others (Table 1). Among statistically significant associations of the largest magnitude were arsenic exposure in drinking water (comparing high to low dose) and various cardiovascular-related outcomes, including CVD (RR 1.32; 95 % CI 1.05, 1.67, *n* = 18) [47]; diabetes type 2 (OR 2.52; 95 % CI 1.69, 3.75, *n* = 6) [48] (this meta-analysis was restricted to populations with high arsenic exposure from Taiwan and Bangladesh), and hypertension (OR 1.27; 95 % CI 1.09, 1.47, *n* = 8) [42].

#### Persistent Organic Pollutants

The POP SRMAs covered residential pesticide exposure, the DDT metabolite DDE, and mixed POPs. Nearly 70 % of reviews evaluated cancers and the remainder other outcomes (Table 1). Among statistically significant associations of largest magnitude, three reviews of residential pesticide exposure in utero and childhood leukemia found pooled ORs of 1.30 (95 % CI 0.86, 1.97, *n* = 4) [53] and 2.05 (95 % CI 1.80, 2.32, *n* = 11) [56] and RR of 2.19 (95 % CI 1.92, 2.50, *n* = 13) [57]. One review found an apparent lack of association between DDE and breast cancer in women (OR 0.97; 95 % CI 0.87, 1.09, *n* = 21) [54].

#### Other Chemicals

The other chemical SRMAs examined exposure to disinfection by-products and fluoride in drinking water and exposure to hair dye. Nearly 70 % of reviews examined cancers, and the remainder involved other outcomes (Table 1). Among observed statistically significant associations of largest magnitude were disinfection by-products and various cancers, including of the bladder (RR 1.21; 95 % CI 1.09, 1.34, *n* = 7; and OR 1.4; 95 % CI 1.2, 1.7, *n* = 8) [62, 66]; rectum (RR 1.4; 95 % CI 1.2, 1.7, *n* = 6; and RR 1.30; 95 % CI 1.06, 1.59, *n* = 10) [62, 64]; and colon (RR 1.27; 95 % CI 1.08, 1.50, *n* = 10) [64]. In addition, one SRMA found a null association between disinfection by-products and both low birth weight (RR 1.0; 95 % CI 0.97, 1.03, *n* = 4) and small for gestational age (RR 1.01 95 % CI 1.00, 1.02, *n* = 6) [60].

### Comparison of Methods to Guidelines

#### Study Background and Goal

As shown in Supplement 2, the vast majority of reviews (94 %) outlined the public health problem and identified a study goal or research question; however, just 54 % defined the goal or question sufficiently clearly to identify all recommended elements (population, health outcome, chemical exposure, and type of study) of a goal statement. Only 40 % of titles self-identified as both SR and MA (an additional 50 % identified as one or the other). One quarter referred to the use of an SRMA guideline; all of these referred either to the PRISMA or MOOSE statements. Only 8 % of reviews reported having an ex-ante study protocol or plan.

#### Search, Selection, and Extraction

Virtually all reviews reported on the electronic databases used to identify studies. Of these, 62 % conducted a wide search (defined as >3 databases); however, in 19 % of reviews, authors limited their search to one database. Medline was the most commonly used database (cited by 96 % of reviews). Most (73 %) reviews reported undertaking hand-searching. An extensive search period (defined as >10 years) was covered in 64 % of reviews. Language was unrestricted in 29 % of reviews, while the grey literature was reported as searched in just 27 % of reviews. Most (80 %) reviews presented key words used in the search or the actual search terms for at least one database. While a large majority (90 %) of reviews cited ex-ante study inclusion or exclusion criteria, less than half (48 %) fully described the screening, text review, and selection processes (though an additional 19 % provided partial description of at least one element of these processes). All relevant study designs were deemed to be included in 70 % of reviews. Efforts to avoid overlapping populations were described in 42 % of reviews. A small minority of reviews excluded studies due to low power (14 %) or poor methods (18 %). A PRISMA-type study selection flow diagram was provided in 38 % of studies.

Study selection and/or data extraction was reported as being done by two or more independent reviewers in over half (54 %) of reviews; in 42 % of SRMAs, no statement was made regarding the number of reviewers. All 48 reviews provided effect point estimates along with measures of variance extracted from underlying studies. In nearly all (92 %) reviews, data on major underlying study covariates were also extracted. In a majority (71 %) of reviews, extracted point estimates were adjusted for commonly reported covariates; however, these often varied across studies. Calculations performed by review authors to derive comparable effect estimates were described in 65 % of reviews. Information on health outcome ascertainment and exposure measurement was extracted by most (79 %) reviews. Authors of underlying studies were contacted for additional information in the case of 27 % of reviews, and 13 % of reviews described using purpose-designed data extraction forms.

#### Methods

The large majority of reviews (94 %) reported using a statistical test of heterogeneity, most commonly the *I*^2^ statistic. Moderate to substantial heterogeneity (e.g., *I*^2^ > 50 %) was found in more than half of SRMAs. Most reviews employed a random-effect model in the presence of observed heterogeneity. Sources of heterogeneity were explored through stratification and/or meta-regression in 71 % of reviews. We assessed health outcomes of underlying studies to be the same or substantially similar (e.g., same ICD code or same definition and ascertainment process) in 83 % of reviews and similar enough to be reasonably compared (e.g., similar definition and ascertainment process) in an additional 13 %. Outcome ascertainment relied predominantly on self-report in nearly one third of reviews.

We assessed exposure metrics to be the same or substantially similar (e.g., the same chemical and measurement technique, with similar exposure conditions) in 31 % of studies and similar enough to be reasonably compared (the same or similar chemical, similar measurement technique, and similar exposure conditions) in an additional 65 % of reviews. In the case of 40 % of reviews, efforts had been made to convert non-similar to similar exposure metrics based on clearly stated assumptions. In addition, based on Blair et al. (1995) our checklist identified four desirable exposure metric characteristics, i.e., that measures preferably be (i) direct (rather than proxy); (ii) individual; (iii) quantitative; and (iv) not based on self-report. Our assessment of exposure measurement characteristics reported in SRMAs was based on the preponderance of evidence (i.e., greater than half of underlying studies meeting the criteria). Results suggest that exposure measures were primarily direct in 44 % of the 48 SRMAs, individual in 52 %, quantitative in 58 %, and avoided self-report in 44 %. Exposure heterogeneity was raised as a risk-of-bias concern in the Discussion sections of 77 % of reviews.

A formal quality and/or risk-of-bias evaluation was reported to have been undertaken in 38 % of reviews, and the results of such evaluations were reported in 27 % of reviews. One quarter of SRMAs reported using a published guideline or checklist (or an author-modified version) for this purpose. Quality scores were used in 21 % of reviews. Publication bias was evaluated in 71 % of reviews, most commonly (over 40 % of reviews) using a funnel chart. Some evidence of publication bias was found in 31 % of reviews.

#### Results, Discussion, and Conclusions

Nearly all reviews presented characteristics of underlying studies in summary tables (96 %), and most illustrated pooled effects using forest plots (85 %). Uncertainty estimates were provided along with summary effect measures in all 48 reviews. Most SRMAs either examined one study design only (or predominantly) or stratified and reported results by study design category. Efforts of underlying studies to examine confounding, case selection, and bias were evaluated and discussed in 58 % of reviews and partially discussed in an additional 21 % of reviews. Thirty-five percent of reviews explored reasons for observed negative or no-effect findings in underlying studies.

Limitations were substantially discussed in 77 % of reviews. Sources of bias and error were raised in this context in 65 % of reviews, while exposure heterogeneity was raised as a limitation in 77 % of reviews. Future EH research recommendations were made in 96 % of reviews. On the other hand, generalizability was discussed in just 33 % of reviews, while EH practice, policy, and improved study-reporting recommendations were made in only 21, 25, and 19 % of reviews.

## Discussion

In this systematic review of EH SRMAs published through mid-2013 comparing methods employed to available guidelines, we found 48 studies using both SR and MA techniques examining associations of low-dose environmental chemicals and adverse health outcomes in general populations. The majority of these reviews were published since 2009, indicating rapid recent uptake of SRMA methods in EH. Nearly half of reviews examined associations between air pollution (indoor and outdoor) exposures and respiratory or CVD outcomes. Over one quarter evaluated associations between various chemical exposures and cancer outcomes. In contrast, we found few SRMAs for many metals (except for arsenic and lead) and POPs, and none for chemicals of more recent interest such as endocrine disruptors. Neurological, immune, and endocrine-related outcomes and upstream effects were also infrequently addressed. Many SRMAs noted that underlying studies were largely from high-income countries (although several covered China only and studies of indoor air pollution were often in developing countries). These findings are broadly consistent with a recent WHO review aimed at estimating the disease burden from chemicals, which found that the largest documentable burden was due to indoor solid fuel smoke and outdoor air pollution (along with secondhand tobacco smoke, not included in our review), but concluded that many chemicals with known health effects could not be included due to lack of meta-analyses providing pooled estimates [2]. High-quality epidemiologic studies and meta-analyses on those missing chemicals are needed in order to more accurately estimate the burden of disease related to environmental exposures.

Reported pooled results were mainly positive and statistically significant. Although some observed associations were not large in magnitude, in many cases strong consistency was noted across numerous studies and dose-response relationships were observed, increasing confidence in the results. Moreover, the risks often involved very large potentially exposed populations: Solid fuels are estimated to be used by half the world’s population; outdoor air pollution affects hundreds of millions of urban dwellers globally, and disinfection by-products and arsenic in water supplies affect millions.

The techniques of SRMA can be useful in synthesizing scientific information provided study goals are well-defined and the methods used are sound [13]. In this review comparing actual use of SRMA methods in EH with available guidelines, we found both strengths and weaknesses. Among strengths, we found a high degree of concordance of SRMAs with many elements of general published guidance such as defining ex-ante selection criteria, reporting health outcome ascertainment data and combining health outcomes that were largely comparable, testing for heterogeneity among studies using a statistical test, appropriately combining using a random-effect model in the presence of heterogeneity, and providing forest plots illustrating pooled effects. Most reviews pursued to some extent unbundling of heterogeneity sources across studies, either through stratification or meta-regression, although in many cases, this was limited. Most discussed some limitations, including causes of heterogeneity, error and bias, and most evaluated publication bias. Nearly all provided recommendations for future research.

However, we also identified reporting and methodological shortcomings in the EH SRMA literature reviewed that we divide into two broad categories: (i) weaknesses with respect to general SRMA reporting guidelines, including up-front problem formulation and implications for generalizability and policy; and in search, selection and extraction methods; and (ii) weaknesses in methods reflecting specificities of EH, including exposure measurement, and evaluation of other quality and risk-of-bias parameters, particularly confounders.

### General SRMA Guidelines

#### Goal Statement and Implications

One of the principle requirements of an SRMA is identification of a well-defined research question, while among key SRMA features are their ability to establish whether findings are consistent and generalizable across populations, and to provide an evidence base for policy development. Some SRMA did provide well-defined goal statements and discuss generalizability, including subsequent relevance for policy and practice. For example, in the context of solid fuel IAP, one review identified at the outset childhood respiratory infection prevention strategies and concluded that, despite the review’s finding of observed heterogeneity in IAP exposure and study designs, consistent mounting evidence warranted their enhanced application [19]. However, we found that the large majority of SRMAs did not do so. Strong, clear goal statements were present in just over half of reviews. Generalizability of findings to other populations, and implications for policy, were discussed in a small minority of reviews. EH SRMAs provide an opportunity for enhancing policy and practice relevance of science findings at a time when the field of EH risk assessment is shifting toward greater attention to up-front problem formulation and its integration with policy- and decision-making [15•, 67]. This opportunity is currently under-utilized.

#### Search, Selection, and Extraction

While searches were generally well reported, their scope was often limited to one or two databases, language was often restricted, and time horizons were in some cases short. Unpublished materials were sought in less than one third of reviews, while research suggests that the grey literature is important in identifying studies [68]. This suggests that some EH SRMAs may be biased toward recent English language studies indexed in Medline. This is also reflected in the finding that approximately half of the reviews that examined publication bias found some evidence of it. Increased use of databases available internationally and eliminating language restrictions (particularly with the goal of identifying studies in low- and middle-income countries) are recommended. In addition, while peer review helps ensure high-quality SRMAs, examination of the grey literature can help identify relevant peer-reviewed papers. Less than half of reviews described procedures to avoid overlapping populations or provided a PRISMA-recommended flow diagram, suggesting some lack of transparency and replicability in study selection. Journal space limitations are likely partly responsible, as such features may have been undertaken and not reported. A solution employed by several SMRAs [34, 36] is providing standard SRMA elements as supplemental material. Nearly half of reviews did not report the recommended two or more independent reviewers while use of piloted extraction forms was reported by a minority of studies, suggesting potential for risk of error in data extraction. This also raises the issue of SRMA resource intensity. Alternatives such as an error-check procedure [20] or a second independent reviewer extracting a random sample of studies [69] may be options to explore. While their validity has yet to be determined, such approaches may help address the trade-off between limited resources and risk of error.

### SRMA Guidelines Specific to EH

#### Exposure Measurement

Exposure characterization was reported to be a key source of between-study heterogeneity in the majority of SRMAs. Although nearly all reviews combined underlying studies whose exposure metrics were largely similar, these metrics were deemed not fully comparable in nearly two thirds of reviews. Exposure metrics commonly used indirect proxies and relied on non-individual measures and self-report, with OAP and metal exposure characterizations more broadly consistent with recommendations than the other three chemical categories. Only a few reviews discussed the likely direction of potential exposure-related error or bias; such insights are useful additions to SRMAs. Some studies not included in this review have concluded that exposure heterogeneity was sufficient to make combining unadvisable, e.g., in the case of risk assessment for PCBs [70]. The lack of updated, widely endorsed EH-specific guidelines for SRMA may be one factor in the observed inconsistency in exposure metrics; a well-disseminated EH SRMA consensus reporting statement with clear principles regarding exposure measurement would be an important contribution. These findings also highlight the potential role for SRMAs in recommending standard practices for exposure measurement protocols in underlying studies. While several SRMAs provided such recommendations [27, 47], this opportunity is under-utilized.

#### Other Sources of Bias

While most reviews extracted data on the covariates adjusted for in underlying studies and reported adjusted rather than crude effect estimates, nearly one quarter did not clearly identify potential confounders, and in most reviews underlying studies adjusted for sets of confounders that differed (e.g., key confounders such as smoking status were often missing). More broadly, quality (standards and reporting) and/or risk-of-bias (internal validity based on study design considerations) evaluations were undertaken in less than 40 % of reviews (though the percentage increased over the time-period examined). Many additional SRMAs examined specific quality- and bias-related issues in their Discussion sections; however, without the structure of a formalized quality evaluation, the analysis may be less transparent, systematic, and thorough. Quality scores, discouraged in guidelines for their potential to add subjective bias, were used in half of quality evaluations undertaken, and 11 % percent of SRMAs reported performing quality assessments but did not report their findings. Journal space constraints may have contributed to the latter outcome, and use of supplemental information files may be a partial solution. As in the case of exposure measurement, clear EH-specific standards for reporting quality and risk-of-bias evaluations would be a substantial contribution.

This is the first study to our knowledge to systematically review the EH SRMA literature comparing methods used in practice with guidance documents. A similar review of SRMAs in the occupational health (OH) field identified 60 OH SRMAs examining mainly cancer outcomes and found limitations and inconsistencies in exposure characterization and inadequate and unclear adjustment for confounders [71], findings generally consistent with ours. Unlike our study, that review found many OH SRMAs used fixed effects models even in the presence of substantial statistically confirmed heterogeneity. A review of SRMA in psychiatric epidemiology found substantial heterogeneity among studies and noted particularly wide variety and poor comparability of outcome measurement instruments [72], while a recent review of risk-of-bias assessments in epidemiological studies found that assessment conclusions were often poorly integrated into study findings [73], both broadly consistent with our results.

Our meta-review is subject to certain limitations. Our search may not have identified all published EH SRMAs during the search period; however, it was designed to cast a wide net and incorporated several strategies to maximize location of all relevant studies. Our quality review checklist was derived from guidelines available during the period of our search. In order to derive a checklist of reasonable length, we left out elements that could have been included; however, we believe that a slightly different list of items is likely to have resulted in similar overall findings. Because the SRMAs in our study covered a wide range of chemical exposures and health outcomes, our findings are at a high level of aggregation; focus on more specific exposure-outcome associations would allow for more in-depth evaluation. One such review of methods and reporting in 16 SRMAs of EH and OH exposures and pregnancy outcomes identified shortcomings primarily related to exposure misclassification and confounding [74], aligning broadly with our findings.

In addition, our study was limited to epidemiological studies in humans. In EH, many decisions are driven by animal-based and cellular-based toxicology experiments, usually in contexts where human studies are limited and/or difficult to carry out. While a few SRMAs reviewed both the toxicology and epidemiology literature [27], the vast majority did not. It was beyond the scope of this review to examine the toxicological literature; however, one such review found that while use of SR methods was adequate, application of MA methods was weak [75]; an updated animal review would be useful in the future, making use of the reporting tools and risk-of-bias criteria currently under development [76].

Our review is broadly consistent with recent efforts at refinement and development of EH SRMA tools by NIEHS/NTP [14•], EPA/IRIS [15•], and the Navigation Guide [17•]. Among the aims of these newer approaches is to go a step beyond evaluating reporting quality to designating a confidence level for EH evidence, based on the strength of inherent underlying study design, with adjustments made for factors that reduce confidence (such as presence of bias in exposure measurement and potential unadjusted confounding) and factors that enhance confidence (such as existence of dose-response relationships, consistency in observed effects, and large magnitudes of effect) [14•]. Our study findings support these efforts by demonstrating gaps and shortcomings in exposure and risk-of-bias assessment in the published literature and by suggesting the contribution to improvements in these areas that could be made by tailored EH SRMA guidelines. It was not the goal of our study to conclude with strength-of-evidence findings for published EH SRMAs; however, use of the emerging tools for this purpose could add to the literature.

## Conclusion

In this meta-review of published EH SRMAs, we found methods and reporting largely conform to available general guidelines. However, there were important exceptions (including in problem formulation, policy implications, and search, selection, and extraction); moreover, we found that SRMAs reflected lower awareness of and poorer conformity with available guidelines applicable to EH studies (particularly exposure measurement and more general risk-of-bias assessment). Many journals have incorporated PRISMA and MOOSE statements into their author guidelines. As a continuation of refinements to SRMA methods for observational studies, we recommend development of a consensus statement of definitive guidelines for EH SRMAs that is published, well-disseminated, and adopted by journals as guidance to study authors and peer reviewers.

Even with the design, methods, and reporting weaknesses observed, the EH SRMAs examined in this review point to broad trends in the EH evidence base, including policy priorities involving large populations (e.g., risk of lung cancer, COPD, and child respiratory infection associated with indoor solid fuel smoke exposure; CVD risks associated with outdoor PM_2.5_ exposure and arsenic); areas of apparent lack of significant associations (e.g., DDE and breast cancer; disinfection by-products, and some birth outcomes); and gaps in the evidence base for certain chemicals and health outcomes (e.g., endocrine disruptors, some metals, and POPs) and populations (e.g., low- and middle-income countries for exposures different from indoor air pollution). We recommend a regularly updated meta-review of the EH epidemiological SRMA literature with the goal of identifying trends in evidence (including strength-of-evidence assessments applying the emerging tools), identifying gaps and research priorities, and stock-taking of methods and reporting quality.

## Electronic supplementary material

ESM 1 (DOCX 35 kb)

ESM 2 (DOCX 31 kb)
