# Supplementary material for: Use of Systematic Review and Meta-Analysis in Environmental Health Epidemiology: a Systematic Review and Comparison with Guidelines
Source: Curr Environ Health Rep. 2015 Jul 3;2(3):272–83. doi: 10.1007/s40572-015-0062-z (PMC4513215; doi:10.1007/s40572-015-0062-z)
Supplement: Supplementary file 1 — (DOCX 35 kb) [file 40572_2015_62_MOESM1_ESM.docx]

**Supplemental file 1: Systematic review of environmental health (EH) epidemiology systematic review and meta-analyses (SRMA): Characteristics of selected studies**

| **SRMA first author** | **Pub Year** | **Chemical** | **Outcome Category** | **Health Outcome** | **Population** | **Region** | **Study design, number in meta-analysis** | **Summary Measure** | **Main reported pooled effect size (95%CI) ^a/^** |
| --- | --- | --- | --- | --- | --- | --- | --- | --- | --- |
| **Indoor Air Pollution (IAP)** | | | | | | | | | |
| Zhao | 2006 | Coal smoke | CANC | Lung | Adults | China | CC 8 | OR | 2.66 (1.39, 5.07) |
| Dherani | 2008 | Solid fuel smoke | RESP | Pneumonia | Children < 5 | International | CC 15 CO 5 CS 3 RTC 1 | OR | 1.78 (1.45, 2.18) |
| Kurmi | 2010 | Solid fuel smoke | RESP | COPD  Chronic bronchitis | Adults | International | CS 4 CC 7 CO 1  CS 8 CC 4 | OR | 2.80 (1.85, 4.00)  2.32 (1.92, 2.80) |
| McGwin | 2010 | Formaldehyde | RESP | Asthma | Children | International | CS 5 CC 4 CO 1 | OR | 1.17 (1.01, 1.36) |
| Hu | 2010 | Biomass smoke | RESP | COPD | Adults | International | CC 4 CS11 | OR | 2.44 (1.79, 3.33) |
| Pope | 2010 | Solid fuel smoke | REPDEV | Low birth weight  Stillbirth | Fetus/Infants | International | CO 2, CS 2, RCT 1  CO1 CC 1 CS 1 | OR | 1.38 (1.25, 1.52)  1.51 (1.23, 1.85) |
| Hosgood | 2011 | Coal smoke | CANC | Lung | Adults | International | CC 25 | OR | 2.15 (1.61, 2.89) |
| Po | 2011 | Biomass smoke | RESP | ARI  Chronic bronchitis  COPD | Children  Women  Women | International | CC 8 CS 2  CC 1 CS 5  CC 2 CS 4 | OR | 3.53 (1.94, 6.43)  2.52 (1.88, 3.38)  2.40 (1.47, 3.93) |
| Misra | 2012 | Solid fuel smoke | RESP | ALRI  LBW | Children < 5 | International | CC 7 CS 2  CO 2 CC 1 CS 2 RTC 1 | OR | 2.51 (1.53, 4.10)  1.45 (1.13,1.87) |
| Kurmi | 2012 | Solid fuel smoke  Coal smoke | CANC | Lung | Adults | Mainly China | CC 28  CC 23 | OR | 1.70 (1.50, 1.94)  1.82 (1.60, 2.06) |
| Sumpter | 2013 | Solid fuel smoke | RESP | Tuberculosis | Adults | International | CC 10 CS 3 | OR | 1.30 (1.04, 1.62) |
| **Outdoor Air Pollution (OAP)** | | | | | | | | | |
| Ward | 2004 | PM10 (/ug/3)  PM2.5 (/ug/3) | RESP | Peak expiratory flow  Peak expiratory flow | Children | International | CO 14  CO 5 | Change | -0.033 (-0.047, -0.019)  -0.144 (-0.243, -0.044) |
| Ito | 2005 | Ozone (/20 ppb) | OTHER | Daily mortality | Adults | International | NS 43 | % excess | 1.6 (1.1, 2.0) |
| Chen | 2008 | PM2.5 (/10ug/3)  NOx (/10/ug/m3  PM2.5 /10ug/m3)  SO2 (/10ug/m3) | OTHER  OTHER  CANC  CANC | Mortality  Cancer  Mortality Lung Cancer | Adults | International | CO 17, CC 20 | RR | 1.06 (1.03, 1.1)  1.08 (1.02, 1.15)  1.21 (1.10, 1.32)  1.07 (0.96, 1.19) |
| Weinmayr | 2010 | PM 10 (/10ug/m3)  NO2 (/10ug/m3) | RESP | Asthma symptoms | Children | International | CO 36  CO 24 | OR | 1.028 (1.006,1.051)  1.031 (1.001, 1.062) |
| Vrijheid | 2011 | NO2 (/10ug/m3)  PM10 (/10ug/m3) | REPDEV | Congenital anomalies | Fetus/infants | International | CC 3 TS 1  CC3 TS 1 | OR | 1.20 (1.02, 1.42)  1.14 (1.01, 1.28) |
|  |  |  |  |  |  |  |  |  |  |
| **SRMA first author** | **Pub Year** | **Chemical** | **Outcome Category** | **Health Outcome** | **Population** | **Region** | **Study design, number in meta-analysis** | **Summary Measure** | **Main reported pooled effect size (95%CI) ^a/^** |
| **Outdoor Air Pollution (OAP), continued** | | | | | | | | | |
| Mustafic | 2011 | CO (ug/m3)  NO2 (/10ug/m3)  SO2 (/10ug/m3)  PM10 (/10ug/m3)  PM2.5 (/10ug/m3)  O3 (/10 ug/m3) | CVD | Myocardial infarction | Adults | International | CCr 11 TS 9  CCr 10 TS 11  CCr 6 TS 8  CCr 7 TS 10  CCr 8 TS 5  CCr 9 TS 10 | RR | 1.048 (1.026, 1.070)  1.011 (1.006, 1.016)  1.010 (1.003, 1.017)  1.006 (1.002, 1.009)  1.025 (1.015, 1.036)  1.003 (0.997, 1.010) |
| Stieb | 2012 | PM10 (/20ug/m3) | REPDEV | Low birth weight | Fetus/infants | International | CO 9 | OR | 1.10 (1.05, 1.15) |
| Pieters | 2012 | PM2.5 (/10ug/m3) | CVD | Heart rate variability | Adults | International | CO 11 CS 2 | % decrease | -2.44 (-3.76, -1.12) |
| Li | 2012 | PM10 (/10ug3)  PM2.5 (/10ug3) | CVD | Stroke | Adults | International | TS 8 CCr 7 | OR | 1.002 (0.999,1.005)  1.006(1.002,1.010) |
| Shah | 2013 | CO (/ppm)  SO2 (/10 ppb)  NO2 (/10 ppb)  PM2.5 (/10ug/m3)  PM10 (/10/m3)  O3 (/10 ppb) | CVD | Heart failure | Adults | International | TS & CCr 18  TS & CCr 14  TS & CCr 18  TS & CCr 10  TS & CCr 22  TS & CCr 18 | RR | 1.018 (1.007, 1.029)  1.014 (1.003, 1.026)  1.009 (1.004, 1.014)  1.016 (1.008, 1.023)  1.010 (1.005, 1.016)  1.001 (0.995, 1.007) |
| Hoek | 2013 | PM2.5 (/10ug/m3) | CVD | CVD mortality | Adults | International | CO 10 | RR | 1.15 (1.04, 1.27) |
| Shang | 2013 | PM2.5 (/10ug3) | OTHER  CVD  RESP | All cause mortality  CVD mortality  Respiratory mortality | Adults | China | TS & CCr 9  TS & CCr 7  TS & CCr 7 | RR | 0.38 (0.31, 0.45)  0.44 (0.33, 0.54)  0.51(0.30, 0.73) |
| **METALS** | | | | | | | | | |
| Pocock | 1994 | Lead (blood) | REPDEV | IQ decline | Children < 5 | International | CS 14 | IQ pts/ doubling | -2.53 (-3.33,-1.73) |
| Navas-Acien | 2006 | Arsenic (water) | OTHER | Diabetes T2 | Adults | Taiwan & Bangladesh | CO 2 CS 4 | OR | 2.52 (1.69-3.75) |
| Chu | 2006 | Arsenic (water) | CANC | Bladder | Adults | International | CC 5 CO 2 | Slope factor | 3 x 10^-5^ |
| Aminzadeh | 2007 | Mercury (amalgam) | OTHER | Multiple sclerosis | Adults | International | CC 3 CO 1 | OR | 1.24 (0.96, 1.61) |
| Navas-Acien | 2008 | Lead (bone) | CVD | Hypertension | Adults | US & Korea | CS 5 | OR | 1.04 (1.01, 1.07) |
| Mink | 2008 | Arsenic (water) | CANC | Bladder | Adults | International | CC 6 CO 2 | RR | 1.11 (0.95, 1.30) |
| Gallagher | 2010 | Cadmium (blood) | CVD | Syst blood pressure  Diast blood pressure | Women | International | CS 3  CS 4 | Regress. Co-effic. | 2.39 (0.69, 4.09)  1.84 (0.95, 2.74) |
| Abhyankar | 2011 | Arsenic (water) | CVD | Hypertension | Adults | International | CS 8 | OR | 1.27 (1.09, 1.47) |
| Moon | 2012 | Arsenic (water) | CVD | CVD | Adults | International | CO 12 CC 3 CS 3 | RR | 1.32 (1.05, 1.67) |
| Rodriguez-Barranco | 2013 | Arsenic (water)  Manganese (blood/hair) | REPDEV | IQ decline | Children < 5 | International | CO 1 CS 4  CS 4 | IQ pts/ doubling | -0.39 (-0.84, 0.06)  -0.7 (-1.07, -0.33) |
| **SRMA first author** | **Pub Year** | **Chemical** | **Outcome Category** | **Health Outcome** | **Population** | **Region** | **Study design, number in meta-analysis** | **Summary Measure** | **Main reported pooled effect size (95%CI) ^a/^** |
| **Persistent Organic Pollutants (POPs)** | | | | | | | | | |
| Priyadarshi | 2001 | Pesticides | OTHER | Parkinson’s disease | Adults | International | CC 14 | OR | 1.85 (1.31, 2.60) |
| Lopez-Cervantes | 2004 | DDE (serum, tissue) | CANC | Breast | Women | International | 21 CC | OR | 0.97 (0.87, 1.09) |
| Allen | 2013 | Pesticides | OTHER | Parkinson’s disease | Adults | International | CC 6 | OR | 1.36 (1.05, 1.75) |
| Turner | 2010 | Resid. pesticides  Resid. insecticides  Resid. Herbicides | CANC | Child leukemia | Women & Fetus/infants | International | CC 11  CS 8  CS 5 | OR | 1.54 (1.13, 2.11)  2.05 (1.80, 2.32)  1.61 (1.20, 2.16) |
| Van Maele-Fabry | 2011 | Resid. Pesticide | CANC | Child leukemia | Women & Fetus/infants | International | CC 13 | RR | 1.74 (1.37, 2.21) |
| Bailey | 2011 | Resid. Pesticide | CANC | Child leukemia | Women & Fetus/infants | International | CC 4 | OR | 1.37 (1.00, 1.88) |
| Wu | 2013 | HCB (blood)  PCBs (blood) | OTHER | Diabetes T2 | Adults | International | CO 4  CO 6 | OR | 2.00 (1.13, 3.53)  1.70 (1.28, 2.27) |
| **Other Chemicals (OTHERS)** | | | | | | | | | |
| Morris | 1992 | Disinf. By-prod. | CANC | Bladder cancer  Rectal cancer | Adults | US | NS 7  NS 6 | RR | 1.21 (1.09, 1.34)  1.38 (1.01, 1.87) |
| Villanueava | 2003 | Disinf. By-prod. | CANC | Bladder cancer | Adults | International | CO 2 CC 6 | OR | 1.4 (1.2, 1.7) |
| Hwang | 2003 | Disinf. By-prod. | REPDEV | Any birth defect | Fetus/infants | International | CS 2 CC 1 | OR | 1.25 (1.11, 1.40) |
| Takkouche | 2005 | Hair dye | CANC | Hemopoietic cancers | Adults | International | CO 9 CC 31 | RR | 1.15 (1.05, 1.27) |
| Nieuwenhuijsen | 2009 | Disinf. By-prod. | REPDEV | Any birth defects | Fetus/infant | International | CC 5 | OR | 1.17 (1.02, 1.34) |
| Rahman | 2010 | Disinf. By-prod. | CANC | Colon cancer  Rectal cancer | Adults | International | CC 7, CO 3  CC8, CO 2 | RR | 1.27 (1.08, 1.50)  1.30 (1.06, 1.59) |
| Grellier | 2010 | Trihalomethane | REPDEV | Low birth weight  Small for gest age | Fetus/infants | International | CO 3 CC 1  CO 6 | OR | 1.0 (0.97, 1.03)  1.01(1.00,1.02) |
| Choi | 2013 | Fluoride | REPDEV | IQ decline | Fetus/infants | Asia | 27 CC | Mean diff. | -0.45 (-0.56, -0.35) |

^a/^ Pooled effect sizes shown are the principal chemical exposure and health effect associations reported in reviews (with stratified results presented when these aid comparison with other reviews).

Abbreviations:

CO: Carbon monoxide; NO2: Nitrogen dioxide; O3: Ozone; PM2.5: Particulate matter < 2.5 microns; PM10: Particulate matter < 10 microns.

CANC: Cancer; CVD: Cardiovascular disease; REPDEV: Reproductive/Developmental; RESP: Respiratory.

CC: Case control; CCr: Case-crossover; CO: Cohort; CS: Cross-sectional; NS: Not stated; TS: Time-series.

OR: Odds ratio; RR: relative risk.
