# Supplementary material for: Use of Systematic Review and Meta-Analysis in Environmental Health Epidemiology: a Systematic Review and Comparison with Guidelines
Source: Curr Environ Health Rep. 2015 Jul 3;2(3):272–83. doi: 10.1007/s40572-015-0062-z (PMC4513215; doi:10.1007/s40572-015-0062-z)
Supplement: Supplementary file 2 — (DOCX 31 kb) [file 40572_2015_62_MOESM2_ESM.docx]

**Supplemental file 2: Systematic review of environmental health (EH) epidemiology**

**systematic review and meta-analyses (SRMA): Results of Quality Review Evaluation** (responses in percent of reviews, out of 48 total)

| **Study rubric** | **Quality Review Checklist Question** | **Y** | **P** | **N** | **ND** | **NA** |
| --- | --- | --- | --- | --- | --- | --- |
| **A. General** | 1. Did title identify study as both SR and M-A (PRISMA #1)? | 40 | 50 | 10 | -- | -- |
|  | 2. Were funding organizations identified (PRISMA #11, #27; MOOSE)? | 67 | 6 | 27 | -- | -- |
| **B. Background** | 3. Was PH problem outlined (PRISMA #3; MOOSE)? | 94 | 4 | 2 | -- | -- |
|  | 4. Was study goal stated as a specific, well-defined research question (PRISMA #4)? | 54 | 40 | 6 | -- | -- |
|  | 5. Was study population identified (MOOSE; Blair)? | 45 | 17 | 38 | -- | -- |
| **C. Methods** | 6. Was existence of a study protocol reported (PRISMA #5)? | 8 | -- | 92 | -- | -- |
|  | 7. Were any SRMA guidelines referenced? | 25 | -- | 75 | -- | -- |
| **- Search** | 8. Was a wide search conducted (PRISMA #7; MOOSE)? | 62 | 19 | 19 | -- | -- |
|  | 9. Were search terms/key words provided (PRISMA #8; MOOSE)? | 80 | 10 | 10 | -- | -- |
|  | 10. Was language unrestricted (PRISMA #6; MOOSE)? | 29 | 8 | 38 | 25 | -- |
|  | 11. Was an extensive search time-period defined (PRISMA #7; MOOSE)? | 64 | 19 | 4 | 13 | -- |
|  | 12. Was hand searching done (MOOSE)? | 73 | -- | 27 | -- | -- |
|  | 13. Were unpublished materials sought (MOOSE; Blair)? | 27 | 2 | 71 | -- | -- |
| **- Selection** | 14. Were clearly-defined *ex-ante* inclusion and/or exclusion criteria reported (PRISMA #6; Blair)? | 90 | 6 | 4 | -- | -- |
|  | 15. Were the selection procedures described (PRISMA #9; MOOSE; Blair)? | 48 | 19 | 35 | -- | -- |
|  | 16. Were all relevant study designs included (MOOSE; Blair)? | 70 | 17 | 13 | -- | -- |
|  | 17. Were overlapping study populations avoided (Blair)? | 42 | -- | 54 | 4 | -- |
|  | 18. Did authors avoid excluding studies due to low power (Blair)? | 86 | -- | 8 | 6 | -- |
|  | 19. Did authors avoid excluding studies due to poor methods (Blair)? | 82 | 4 | 8 | 6 | -- |
| **- Extraction** | 20. Was data extraction done by two or more reviewers (PRISMA #10)? | 54 | 2 | 2 | 42 | -- |
|  | 21. Were purpose-designed extraction forms described and/or piloted (PRISMA #10; Blair)? | 13 | 17 | 70 | -- | -- |
| **Study rubric** | **Quality Review Checklist Question** | **Y** | **P** | **N** | **ND** | **NA** |
|  | 22. Were extracted summary statistics adjusted for confounders (PRISMA #11; Blair)? | 71 | 17 | -- | 6 | -- |
|  | 23. If effect estimates were calculated was this noted and methods provided (PRISMA #11; Blair)? | 65 | 2 | 33 | -- | -- |
|  | 24. Were other relevant data extraction categories described (PRISMA #11)? | 92 | 4 | 4 | -- | -- |
|  | 25. Was information on determination of health outcomes extracted (PRISMA #11)? | 79 | 8 | 13 | -- | -- |
|  | 26. Was information on exposure measurement method extracted (PRISMA #11)? | 79 | 8 | 13 | -- | -- |
|  | 27. Were authors contacted for additional data (PRISMA #11; MOOSE; Blair)? | 27 | -- | 71 | 2 | -- |
| **- Quality/Bias** | 28. Was an evaluation of study quality/risk of bias done (PRISMA #12; MOOSE; Blair)? | 38 | 2 | 60 | -- | -- |
|  | 29. Was a standard guideline for developing the study quality review reported? | 25 | -- | 19 | -- | 56 |
|  | 30. Were efforts of underlying studies to examine confounding evaluated (MOOSE)? | 58 | 21 | 21 | -- | -- |
|  | 31. Was use of quality scores avoided (MOOSE; Blair)? | 77 | -- | 21 | -- | 2 |
|  | 32. Was publication bias assessed (PRISMA #15)? | 71 | 4 | 25 | -- | -- |
| **- Heterogeneity** | 33. Was heterogeneity tested for with a statistical test (PRISMA #13; MOOSE; Blair)? | 94 | 4 | 2 | -- | -- |
|  | 34. Was a random effects model used if heterogeneity warranted (PRISMA #13; MOOSE; Blair)? | 94 | 2 | 4 | -- | -- |
|  | 35. Were sources of heterogeneity explored through stratification or regression (PRISMA #16; Blair)? | 71 | 13 | 17 | -- | -- |
| **- Exposure** | 36. Were exposure metrics the same or comparable across studies (Blair)? | 31 | 65 | -- | 4 | -- |
|  | 37. Were non-similar measures converted to common metrics before combining (Blair)? | 40 | 2 | 58 | -- | -- |
| **Study rubric** | **Quality Review Checklist Question** | **Y** | **P** | **N** | **ND** | **NA** |
|  | 38. Were exposure measures direct (Blair)? | 44 | 17 | 35 | 4 | -- |
|  | 39. Were exposure metrics individual (Blair??)? | 52 | 8 | 38 | 2 | -- |
|  | 40. Were exposure measures quantitative (Blair)? | 58 | 15 | 23 | 4 | -- |
|  | 41. Did exposure measures avoid self-report (Blair??)? | 54 | 6 | 38 | 2 | -- |
| **- Outcome** | 42. Was health outcome the same or comparable across studies (Blair)? | 83 | 13 | -- | 4 | -- |
|  | 43. Did health outcome ascertainment avoid self-report (Blair)? | 67 | 13 | 17 | 4 | -- |
| **D. Results** | 44. Was a flow diagram summarizing selection of studies included (PRISMA #17; MOOSE; Blair)? | 38 | -- | 63 | -- | -- |
|  | 45. Was a table of descriptive data by included study provided (PRISMA #18; MOOSE; Blair)? | 96 | 2 | 2 | -- | -- |
|  | 46. Were measures of uncertainty reported with effect estimates (PRISMA #20; MOOSE; Blair)? | 100 | -- | -- | -- | -- |
|  | 47. Were the results of the quality/risk of bias review reported (PRISMA #19; MOOSE; Blair)? | 27 | -- | 10 | -- | 63 |
|  | 48. Was evidence of publication bias found (PRISMA #15; MOOSE)? | 31 | 6 | 40 | -- | 23 |
|  | 49. Was stratification done by factors identified in the quality analysis (PRISMA #16; MOOSE; Blair)? | 21 | 4 | -- | -- | 75 |
|  | 50. Did stratification to reduce heterogeneity modify pooled effect size (MOOSE; Blair)? | 48 | 4 | 25 | -- | 23 |
|  | 51. If studies found negative or no-effect results were reasons explored (Blair)? | 35 | 4 | 60 | -- | -- |
|  | 52. Was a forest plot for summary effect estimate provided (PRISMA #20; MOOSE; Blair)? | 85 | -- | 15 | -- | -- |
|  | 53. Did final effect size reflect quality and heterogeneity sensitivity analysis (PRISMA #16; Blair)? | 69 | 2 | 23 | -- | 6 |
| **E. Discussion** | 54. Were limitations discussed (PRISMA #25; MOOSE)? | 77 | 13 | 10 | -- | -- |
|  | 55. Were sources of bias and error discussed (PRISMA #15; MOOSE)? | 65 | 15 | 21 | -- | -- |
|  | 56. Was exposure heterogeneity raised as a concern (Blair)? | 77 | 6 | 17 | -- | -- |
|  | 57. Was generalizability discussed (MOOSE)? | 33 | -- | 67 | -- | -- |
| **Study rubric** | **Quality Review Checklist Question** | **Y** | **P** | **N** | **ND** | **NA** |
| **F. Conclusions** | 58. Were environmental health research recommendations discussed (PRISMA #24, #26; MOOSE)? | 96 | -- | 4 | -- | -- |
|  | 59. Were public environmental health practice recommendations discussed (PRISMA #24)? | 21 | -- | 79 | -- | -- |
|  | 60. Were public environmental health policy recommendations discussed (PRISMA #24)? | 25 | 6 | 69 | -- | -- |
|  | 61. Were recommendations for improved study reporting provided? | 19 | -- | 81 | -- | -- |

Notes:

This checklist was developed based on reporting guidelines for SRMAs available to researchers during the review period (through mid-2013), including the PRISMA (Preferred Reporting Items for Systematic Reviews and Meta-Analyses) Statement (Moher et al. 2009), the MOOSE (Meta-Analysis of Observational Studies in Epidemiology consensus statement (Stroup et al. 2000), and guidelines for use of SRMA in environmental epidemiology resulting from a 1994 workshop (Blair et al. 1994).

As indicated above, checklist items for the general features of SRMA were based primarily on the PRISMA and MOOSE statements, while those related to quality and risk-of-bias evaluation, heterogeneity testing, exposure measurement and outcome ascertainment were also based on Blair et al.

Checklist questions were designed to have up to five possible responses: yes (Y), consistent with guideline; partial (P), in some part consistent with guideline; no (N), inconsistent with guideline; cannot determine based on data (ND); and not applicable to study (NA). A set of agreed definitions for each possible response was determined ex-ante for all questions to facilitate consistent analysis between reviewers.
